# The Importance of Memory Specificity and Memory Coherence for the Self: Linking Two Characteristics of Autobiographical Memory

**DOI:** 10.3389/fpsyg.2017.02250

**Published:** 2017-12-22

**Authors:** Elien Vanderveren, Patricia Bijttebier, Dirk Hermans

**Affiliations:** ^1^Center for the Psychology of Learning and Experimental Psychopathology, KU Leuven, Leuven, Belgium; ^2^School Psychology and Development in Context, KU Leuven, Leuven, Belgium

**Keywords:** autobiographical memory, memory specificity, memory coherence, self, Self-Memory System

## Abstract

Autobiographical memory forms a network of memories about personal experiences that defines and supports well-being and effective functioning of the self in various ways. During the last three decades, there have been two characteristics of autobiographical memory that have received special interest regarding their role in psychological well-being and psychopathology, namely memory specificity and memory coherence. Memory specificity refers to the extent to which retrieved autobiographical memories are specific (i.e., memories about a particular experience that happened on a particular day). Difficulty retrieving specific memories interferes with effective functioning of the self and is related to depression and post-traumatic stress disorder. Memory coherence refers to the narrative expression of the overall structure of autobiographical memories. It has likewise been related to psychological well-being and the occurrence of psychopathology. Research on memory specificity and memory coherence has developed as two largely independent research domains, even though they show much overlap. This raises some important theoretical questions. How do these two characteristics of autobiographical memory relate to each other, both theoretically and empirically? Additionally, how can the integration of these two facilitate our understanding of the importance of autobiographical memory for the self? In this article, we give a critical overview of memory specificity and memory coherence and their relation to the self. We link both features of autobiographical memory by describing some important similarities and by formulating hypotheses about how they might relate to each other. By situating both memory specificity and memory coherence within Conway and Pleydell-Pearce’s Self-Memory System, we make a first attempt at a theoretical integration. Finally, we suggest some new and exciting research possibilities and explain how both research fields could benefit from integration in future research.

## Introduction

Research has consistently demonstrated the intricate relationship between autobiographical memory and the self. Different features of autobiographical memories contribute to well-being and effective functioning of the self in various ways and some of these memory characteristics show some overlap. In this paper, we will focus on two features of autobiographical memories in particular: memory specificity and memory coherence. Research on these two features developed rather independently throughout the past three decades. This is, in our opinion, quite surprising since there seem to be similarities between both when comparing both literatures. Memory specificity and memory coherence show, for example, similar associations to well-being and psychopathology. They also show a quite similar developmental pathway throughout childhood and adolescence. These similarities make us wonder how they might relate to each other and how they both relate to the self. Integrating both research domains offers, in our opinion, new and exciting possibilities for future research. The goal of this paper is therefore to point out the exciting research possibilities that can come from integrating coherence and specificity and explain why and how both research fields could benefit from such integration in future research. First, we will focus on the relationship between autobiographical memory and the self, after which we will give a brief overview of the literature on memory specificity and memory coherence and how they relate to the self. We will also point to some remaining questions and limitations within both research fields. Then, we will link both features of autobiographical memory by describing some important similarities and by formulating hypotheses about how they might relate to each other. We will make a first attempt at a theoretical integration by positioning both memory specificity and memory coherence within Conway and Pleydell-Pearce’s Self-Memory System. Finally, we will suggest some new and exciting research possibilities and we will describe in more detail how integrating both in future research could be beneficial for research on both memory specificity and memory coherence. Throughout this paper, we will refer to Conway and Pleydell-Pearce’s Self-Memory System (Conway and Pleydell-Pearce, 2000) to illustrate the reciprocal and intricate relationship between autobiographical memory and the self. Although the Self-Memory System is just one of many available theoretical models, we have chosen this model because it offers an interesting theoretical framework to look at how memory specificity and memory coherence could potentially be integrated.

## Autobiographical Memory and the Self

Autobiographical memories are memories about personally experienced events that go beyond the mere factual description of the event to include personal beliefs, thoughts, and emotions (Bruner, 1990; Fivush, 2010). Together they form a network of memories called autobiographical memory, which contains personal information that make up a person’s unique life story (Fivush et al., 2011). The structure of this integrative network is illustrated in Conway and Pleydell-Pearce’s Self-Memory System (Conway and Pleydell-Pearce, 2000; Conway et al., 2004). An individual’s personal life story consists of different layers of autobiographical memories that are represented in the autobiographical knowledge base and the episodic memory system: life story schema, lifetime periods, general events, and event-specific knowledge. These categories are ordered in a hierarchy going from general to specific personal information. Life story schema is a mental representation of major components of a person’s life and represents the individual’s understanding of how one’s life story is constructed within the culture one lives in. Lifetime periods represent certain periods in a person’s life with a clear beginning and ending that have distinctive thematic features (e.g., my time at the University of Leuven). General events are more specific than lifetime periods and are composed of clusters of repeated events or sequences of related events that share a certain theme (e.g., driving to class in the mornings). Accessing one memory of a general event triggers others that are comprised within the same or related clusters. Finally, event-specific knowledge refers to perceptual and sensory-rich information about single personal experiences (e.g., my thesis defense). Autobiographical memories are retrieved through the consecutive activation of these three categories, with more general information at the top layers triggering related information at the more specific levels (Conway and Pleydell-Pearce, 2000; Conway et al., 2004). Together, the personal information that is stored in these different hierarchical layers represents who we are, were, and can be in the future (Conway, 2005).

Autobiographical memory supports well-being and effective functioning of the self in different ways. Before we describe the role of autobiographical memory for the self in more detail, we will take a closer look at how the self can be conceptualized. In the Self-Memory System of Conway and Pleydell-Pearce, the self is represented by three separate constructs that interact with each other: the working self, the conceptual self and the long-term self (see Conway and Pleydell-Pearce, 2000; Conway et al., 2004). The working self consists of a hierarchy of current personal goals that are thought to guide cognitions, emotions, and behavior toward achieving these goals. Personal experiences are organized and evaluated in function of their correspondence to current goals. The result of this evaluation determines which experiences will be more likely to be encoded and later recalled by favoring personal events that are in accordance to one’s goals. The long-term self contains more permanent information about the self that is required for the working self to organize and evaluate personal experiences. One aspect of the long-term self is the conceptual self, which consists of attitudes, values, beliefs, relational schemas etc. Besides the conceptual self, the long-term self also includes the autobiographical knowledge base, which we discussed earlier. The working self, conceptual self, and long-term self interact and by doing so, influence the formation of autobiographical memories.

The previous seems to indicate that autobiographical memory and the self share an intricate and reciprocal relationship. The self mediates the formation of autobiographical memories and controls their accessibility. Conversely, autobiographical memory serves the self in many different ways (see Bluck and Alea, 2002; Fivush, 2011; or Pillemer, 1992 for a more detailed overview). First of all, autobiographical memories provide a sense of continuity through time for the individual; the idea that one is the same person now as in the past and will be in the future. Even if one changes, these changes are explained and understood through experiences of growth that lead to new perspectives on self. Second, autobiographical memory facilitates the creation and maintenance of a social network through reminiscing with others. Third, memories about past personal experiences guide our current and future behavior. Fourth and final, by constructing memories about negative or stressful experiences and integrating these memories within a broader framework, autobiographical memory facilitates the process of coping with and resolving negative emotions (Fivush, 2011). These self-serving functions of autobiographical memory all contribute to well-being (Fivush, 2011).

Single autobiographical memories consist of different features, like the amount of detail they entail, their emotional intensity, structural characteristics, etc. Research on how specific characteristics of personal memories contribute to well-being enhances our understanding of how autobiographical memory relates to the self. In this paper, we take a closer look at two particular features of autobiographical memories and how they both relate to the self: memory specificity and memory coherence.

## Memory Specificity

During the last three decades, one particular characteristic of autobiographical memory, namely memory specificity, has been an important topic of research. Memory specificity is usually operationalized as the ability to retrieve specific personal memories following emotional cue words. For a personal memory to be considered specific, it has to consist of a particular event that happened at a particular time and place and did not last longer than 1 day (Williams and Broadbent, 1986). The whole memory specificity literature can be brought back to a rather coincidental observation by Williams and Broadbent (1986). They found that, compared to a healthy control group, depressed and suicidal individuals showed difficulty retrieving specific personal memories following emotional cue words (a paradigm that is now commonly referred to as the Autobiographical Memory Test or AMT). Instead of retrieving specific memories as instructed, they responded more frequently with overgeneral memories such as categorical memories (i.e., the memory refers to a class of generic events) or extended memories (i.e., the described event lasted longer than 1 day) (Williams and Broadbent, 1986). This phenomenon is referred to as Overgeneral Autobiographical Memory or OGM. When asked to recall a specific memory following the cue-word ‘happy,’ someone who has a tendency to recall overgeneral memories would say for instance ‘Whenever I’m on vacation’ instead of giving a more specific answer like ‘I felt happy last Saturday night when I went to the movies with my best friend.’

As we mentioned earlier, autobiographical memory serves the self in four different ways; it helps us guide future behavior, form and maintain a social network, create a continuous sense of self and cope with negative emotions and experiences (Pillemer, 1992; Bluck and Alea, 2002; Fivush et al., 2003; Fivush, 2011). These four functions all have been found to relate to psychological and physical well-being (Fivush, 2011). To explain the importance of the specificity of autobiographical memories for the self, we consecutively describe the way memory specificity is involved in these four functions.

### Self-Guidance

Memory specificity supports effective functioning of the self by guiding current and future behavior. For instance, when faced with a problem, being able to recall a specific past event facilitates problem solving since knowledge about past solutions can be transferred to the current situation. In other words, being able to recall a specific past experience that is similar to the current situation and the details about how the past situation was dealt with enhances problem solving of the current situation (Evans et al., 1992; Goddard et al., 1996, 1997; Scott et al., 2000; Raes et al., 2005). Difficulty recalling specific memories therefore hinders problem solving. Additionally, difficulty remembering specific past experiences also relates to difficulty picturing specific future events and setting goals for the future (Belcher and Kangas, 2014). Not being able to imagine specific future happenings has been found to evoke feelings of hopelessness (Evans et al., 1992; Williams et al., 1996).

### Self-in-Relation

Sharing autobiographical memories with others facilitates the formation and maintenance of a social network. It is assumed that people develop close and intimate relationships with others by sharing specific personal memories (Alea and Bluck, 2003). In order to really get to know someone, sharing specific personal memories is important, since these memories reveal the person’s unique life story (e.g., unique adventures or obstacles they encountered throughout their life) (Habermas and Bluck, 2000). By doing this, the individual differentiates oneself from others (Beike et al., 2016). Research has indeed shown that sharing specific personal memories increases intimacy and closeness. People report feeling more intimate to the conservation partner and experiencing a more positive mood after conversations in which specific memories were shared (e.g., Pasupathi and Carstensen, 2003; Alea and Bluck, 2007). This relationship between intimacy and sharing specific memories appears to be bidirectional, since feeling close to someone facilitates sharing specific personal memories. Feelings of intimacy and closeness create a safe context to disclose specific personal information, which in turn increases intimacy (Beike et al., 2016). However, a recent study by Beike et al. (2016) has shown that sharing specific personal memories does not increase intimacy and closeness more than disclosing general personal information does. So, the act of sharing personal information in itself appears to be more important for creating close relationships with others than the nature (specific versus general) of the information.

### Self-Identity

Besides guiding future behavior and facilitating social interactions, memory specificity is also involved in creating a stable sense of self. Autobiographical memory represents someone’s unique personal history that defines who one is across time. Recalling specific personal experiences plays an important role in creating a continuous sense of self (Bluck and Habermas, 2000), which is considered a crucial developmental task during adolescence (Erikson, 1968; McAdams, 1985). By interpreting, evaluating, and linking together different specific personal experiences that are believed to be significant to understand who one is, a sense of self evolves. This refers to the process of autobiographical reasoning, which facilitates identity development (Bluck and Habermas, 2000; Habermas and Bluck, 2000; Fivush et al., 2011). However, studies directly examining the influence of memory specificity on identity development are rather scarce. It is, nonetheless, reasonable to assume that difficulty recalling specific personal experiences would have an influence on one’s sense of self. If someone is unable to recall specific memories that are unique to the person and differentiate oneself from others, it is plausible to assume that this would be associated with a less profound and stable sense of self. Some studies on autobiographical memory in patients with Alzheimer’s disease and certain personality disorders seem to support this hypothesis. Alzheimer’s disease is for example characterized by deficits in recalling personal memories (including specific memories), which leads to an impaired sense of self and identity (see El Haj et al., 2015). Additionally, there seems to be a negative association between identity confusion and memory specificity in patients with Borderline Personality Disorder (Van den Broeck, 2014), and patients with Dissociative Identity Disorder show signs of overgeneral autobiographical memory as well (Huntjens et al., 2014). Research specifically addressing the influence of overgeneral autobiographical memory on sense of self and identity development would be a valuable addition to these findings.

### Self-Regulation

Being able to recall specific personal memories contributes to aspects of psychological well-being and effective functioning of the self, as demonstrated earlier. Additionally, research has shown that overgeneral autobiographical memory contributes to the development of psychological problems like depression and post-traumatic stress disorder (see Sumner et al., 2010 or Williams et al., 2007 for reviews). When individuals experience negative emotions or events, they have to regulate and resolve them in order to move on. If these self-regulatory processes are insufficient or maladaptive, the individual is at risk to develop psychological problems. Different emotion regulation strategies relate to the presence of overgeneral autobiographical memory and can explain the relationship between overgenerality and psychopathology, which we will discuss in more detail later on. First, we take a closer look at the association between overgeneral autobiographical memory and psychopathology.

Research has shown that patients who suffer from depression or PTSD have more difficulty recalling specific memories following emotional cues than healthy controls. More specifically, studies have shown that overgeneral autobiographical memory can predict the course and onset of depression over and above initial depressive symptoms (Sumner et al., 2010). This difficulty retrieving specific memories remains present whilst in remission. In other words, overgeneral memory appears to be a steady characteristic of depressed and formerly depressed individuals (see Williams et al., 2007 or Sumner et al., 2010 for a review). This tendency to recall general rather than specific memories has likewise been observed in individuals suffering from PTSD. Patients who suffer from PTSD show difficulty retrieving specific memories related to the trauma they experienced and over time this difficulty expands to unrelated memories as well (e.g., Kuyken and Brewin, 1995; Williams et al., 2007). Additionally, overgeneral autobiographical memory is able to predict PTSD diagnosis following a traumatic experience (Kleim and Ehlers, 2008).

It is commonly assumed that overgeneral memory is not a general characteristic of psychopathology, but that it is specific to affective disorders like depression and PTSD (Williams et al., 2007). Patients who suffer from generalized anxiety disorder, OCD, or specific phobias for example do not exhibit difficulty retrieving specific memories (Burke and Mathews, 1992; Wilhelm et al., 1997; Wessel et al., 2001; Wenzel et al., 2002, 2003). The same applies to eating disorders (e.g., Dalgleish et al., 2003) and personality disorders like borderline personality disorder (e.g., Van den Broeck et al., 2015). When overgeneral memory is observed in other psychological disorders, it can usually be explained by comorbid depressive symptomatology (Sumner et al., 2010; but see Ridout et al., 2015).

Different mechanisms can explain the development of overgeneral autobiographical memory. Williams and colleagues (Williams, 2006; Williams et al., 2007) developed a theoretical model (CaR-Fa-X model) that focuses on three underlying mechanisms that are thought to explain the development of overgeneral memory and how it relates to psychopathology; rumination, functional avoidance, and impaired executive functioning. When trying to recall a specific event, an individual will start his search at the most general level of autobiographical memory and carry on to the level of event-specific knowledge. Different mechanisms can interrupt this search, whether or not in interaction with each other, and lead to overgeneral memory. Ruminating, or continuously dwelling over one’s sad or depressive feelings (Nolen-Hoeksema, 1991), can disrupt the search for specific memories. When searching for a specific memory, an individual with a negative self-concept could activate a general memory about him- or herself that triggers ruminative thinking about the memory and the personal value of it. While ruminating about this general memory, the individual will not continue the search for a specific memory. Repeated ruminating can result over time in the development of overgeneral autobiographical memory. Besides rumination, functional avoidance of the potentially negative and overwhelming emotions memories can evoke, also plays a role in the development of overgeneral memory. By avoiding to recall specific personal experiences, intentional or otherwise, one protects oneself from the associated emotions. By not being confronted with the potential negative and painful emotions related to the memory, the avoidant behavior will be negatively reinforced, increasing the likelihood of avoiding specific memories in the future. The avoidant behavior can generalize to other memories and eventually lead to overgeneral memory. So, the functional avoidance mechanism could be seen as a means of affect regulation that could be beneficial in the short term, but maladaptive over time. Finally, the search from general lifetime periods to event-specific knowledge requires sufficient executive capacities. When asked to recall a specific memory, the individual has to be able to inhibit non-related autobiographical knowledge and hold the final result of the search in his or her working memory. Affective disorders like depression are often characterized by reduced executive functioning, which hinders the search.

The role these mechanisms play in the development of overgeneral autobiographical memory has been repeatedly studied over the past two decades. Although several studies support the claims and predictions of the CaR-Fa-X model, there are studies that fail to replicate these findings (see Valentino, 2011; Sumner, 2012; or Williams et al., 2007 for reviews).

### Summary and Discussion

More than three decennia of research on memory specificity has demonstrated the important role memory specificity plays in psychopathology and the mechanisms that underlie this relationship. To recapitulate, individuals suffering from depression and PTSD have been found to experience difficulty recalling specific personal memories compared to healthy controls. It has been theorized that three different mechanisms are responsible for the development of overgeneral autobiographical memory; rumination, functional avoidance and impaired executive capacities. However, the field of memory specificity is not without limitations. Since the original observation by Williams and Broadbent (1986), different research labs started focusing on this surprising cognitive phenomenon by implementing the same emotional cueing design, which is now commonly referred to as the Autobiographical Memory Test (AMT). The memory specificity literature is therefore originally based upon one particular paradigm instead of theoretical considerations. This paradigm-driven nature of the memory specificity literature created methodological benefits by facilitating comparisons between studies from different research groups, but it also raised some important theoretical questions. The most important question, in our opinion, being what the AMT actually measures. How Williams and Broadbent originally operationalized specific memories in the instructions of the AMT as being “memories about a particular event that happened on a particular day and did not last longer than 1 day” does not correspond to the way specific memories are defined in the Self-Memory System (Conway and Pleydell-Pearce, 2000). Conway and Pleydell-Pearce define specific memories or ‘event-specific knowledge’ as detailed information about individual events that are particularly rich in perceptual and sensory information. No limitations about the duration of the event are imposed. More research seems necessary to examine what the AMT exactly measures and how this can best be fitted within the Self-Memory System of Conway and Pleydell-Pearce.

## Memory Coherence

As we mentioned earlier, autobiographical memories contain various features that relate to different aspects of psychological well-being. In addition to their specificity, autobiographical memories also differ in their overall internal structure, i.e., memory coherence. In this paper, we depart from the multidimensional model developed by Reese et al. (2011). This model states that personal memories can be considered coherent if three conditions are met; (1) the memory contains information about when and where the events took place, (2) the events are described in a logical and chronological order and (3) the memory consists of a highpoint and resolution, along with affective and evaluative information (Reese et al., 2011). These criteria show that memory coherence goes beyond the mere structural aspects of autobiographical memories to include a qualification of the emotional representation of the events. These three conditions of memory coherence represent three separate subcomponents or dimensions; respectively contextual, chronological and thematic coherence (Reese et al., 2011). Memory coherence is usually assessed by asking individuals to narrate about a personal experience (either orally or on paper), after which these narratives are coded for their coherence by an independent rater using one of the available coding schemes. Similar to memory specificity, memory coherence is related to well-being and effective functioning of the self in various ways. In what follows, we describe how memory coherence is associated with the self, but first we illustrate the concept a bit more with an example.

“The 50th wedding anniversary of my grandparents about 6 months ago was definitely one of the happiest moments of my life. They threw a big party at their house. When we arrived, we all got a copy of their original wedding picture with the schedule of the evening on the back. They renewed their vows during a ceremony in the garden, which is next to a beautiful lake. It looked so beautiful and romantic! My grandpa gave a beautiful speech, which made me very emotional. Afterward, we all had dinner and my grandparents reminisced about their lives together. I’ve always been a firm believer of true love, but a couple of months before my grandparents’ anniversary, I went through a tough break-up. This made me kind of pessimistic and doubtful. Seeing my grandparents in love after all those years made me realize that true love is out there and that I should continue looking for it.”

This narrative can be considered coherent across all three dimensions. By describing exactly when and where the event took place, the individual creates a contextual coherent account of the experience. By using words like ‘first,’ ‘then’ and ‘afterward,’ the sequence of the events becomes clear. When referring to a previous break-up and stating when this took place relative to the anniversary, the individual places the experience within a broader timeframe, making the narrative chronologically coherent. Finally, the narrative contains a subjective evaluation of the experience and ends with a resolution, which contributes to the thematic coherence of the narrative.

### Self-Guidance

Memory coherence facilitates emotional problem solving through the process of meaning making. Narratives reflect the way in which we make sense of the world and our experiences (Bruner, 1990; McAdams, 2001). By creating coherent narratives about past experiences we can create meaning. This is particularly important when faced with negative or stressful experiences. Creating coherent narratives about negative or stressful events makes it possible to express and regulate the related thoughts and emotions and, eventually, come to some sort of resolution or closure (Pennebaker, 1997; Fivush and Baker-Ward, 2005). This facilitates recovery and is beneficial for the effective functioning and well-being of the self. One could then utilize this previous experience for future occurrences However, creating coherent narratives about negative events can be challenging, since the related emotions can be overwhelming. As research on this topic has shown, narratives about negative events are generally more coherent than narratives about positive or neutral events (Fivush et al., 2008). This could be explained by the fact that negative or stressful experiences imply a problem that must be solved, which in turn may lead to more efforts to construct coherent narratives about the events in order to understand and create meaning out of them (Fivush et al., 2008). So, memory coherence facilitates emotional problem solving and should be considered an important part of the meaning making process, because it represents the extent to which people can explain and understand the events they have experienced (Fivush et al., 2008).

### Self-Identity

As we mentioned earlier, creating a stable and continuous sense of self is key to a healthy identity development, which is an important developmental task during adolescence (Erikson, 1968; McAdams, 1985). Akin to memory specificity, memory coherence is involved in forming a personal identity. Through the process of autobiographical reasoning (i.e., interpreting, evaluating, and integrating different specific personal experiences that are believed to be significant to understand who one is), single narratives will be connected and integrated into an overall life story (Habermas and Bluck, 2000; Habermas and de Silveira, 2008). An individual’s life story is a subjective representation of his or her personal and unique development (Habermas and de Silveira, 2008). It’s important that this life story is coherent and not merely a bundle of single narratives, since forming a coherent overall life story leads to feelings of purpose and meaning in life, and results in the formation of a continuous sense of self or personal identity (Kernberg, 1984; Antonovsky, 1985; McAdams, 1985; Habermas and de Silveira, 2008).

### Self-in-Relation

Besides facilitating emotional problem solving and creating a continuous sense of self, memory coherence is also involved in the social function of autobiographical memory. Narrating and reminiscing about personal experiences is a highly social activity that facilitates the creation and maintenance of a social network. Through reminiscing with others our lives become intertwined and we create a shared past (Fivush et al., 2006). The manner in which this reminiscing occurs, especially in the context of mother-child reminiscing, relates to the child’s memory coherence and well-being (see Fivush et al., 2006 for a review). Mothers who are more coherent will be more elaborative when reminiscing with their children (Reese, 2008). This elaborative style of reminiscing about personal experiences, especially about negative or stressful experiences, is related to numerous adaptive outcomes (Fivush et al., 2006). Besides being more coherent when reminiscing with others later in life (e.g., Fivush and Fromhoff, 1988), children from elaborative mothers also tend to have a more coherent and consistent self-concept (Welch-Ross et al., 1999; Bird and Reese, 2006) and a more advanced emotional understanding (Laible, 2004). Additionally, these children possess more effective coping styles and show less internalizing and externalizing symptoms (Sales and Fivush, 2005; Fivush and Sales, 2006).

### Self-Regulation

As we mentioned earlier, creating coherent narratives about personal experiences facilitates emotion regulation (Pennebaker, 1997; Fivush and Baker-Ward, 2005). This is particularly important when faced with negative or stressful experiences, since such experiences can make the individual vulnerable for developing various psychological problems (e.g., Cole et al., 1990; Kessler, 1997; Kendler et al., 2003). Keeping this in mind, it is not surprising that memory incoherence is related to the presence of psychopathology. Individuals who experience difficulty with narrating about personal experiences in a coherent manner show more internalizing as well as externalizing symptoms. More specifically, memory incoherence has been associated with depressive symptoms, behavioral problems, and PTSD (e.g., Foa et al., 1995; von Klitzing et al., 2000; von Klitzing et al., 2007; Müller et al., 2014; Stadelmann et al., 2015). Additionally, a pattern of incoherence has been observed in patients suffering from a borderline personality disorder, eating disorder, and obsessive-compulsive disorder (Rasmussen et al., 2017). Research also seems to suggest that memory coherence can act as a buffer against the impact of negative life experiences. As we discussed earlier, memory coherence enables creating meaning out of a negative or stressful event and regulating the related cognitions and emotions. As a result, it can be argued that memory coherence protects the individual from the damaging effects negative life experiences can have on well-being, making incoherent persons more vulnerable (Müller et al., 2014; Stadelmann et al., 2015). However, there are still some outstanding questions and inconsistencies regarding the association between memory coherence and psychopathology, which we will discuss in more detail below.

Research on the association between memory coherence and internalizing symptoms yielded inconsistent findings. Stadelmann (2006, Unpublished), for example, found no relation between a lack of memory coherence and the presence of internalizing symptoms. Two possible explanations can be formulated for these discrepancies. First, internalizing symptoms form a very heterogeneous group of symptoms which contains, for example, both depressive and anxiety-related symptoms. It is possible that memory coherence is only related to one of these clusters of symptoms, which could explain the inconsistent findings (Stadelmann et al., 2015). A related explanation is the fact that the majority of studies used broadband screening instruments (i.e., instruments that look at a variety of psychological symptoms instead of focusing on a specific disorder) that do not differentiate between different types of internalizing symptoms, which may contribute to the inconsistent findings.

The association between memory coherence and PTSD has also been topic of intense debate over the past few years (see Brewin, 2016; Rubin et al., 2016a,b). Different theories postulate that PTSD is characterized by memory incoherence. This means that when PTSD patients are asked to recall the trauma they experienced, their memories of this event will be more fragmented and disorganized, as compared to non-related memories and healthy control groups. In accordance with this hypothesis, numerous studies report more incoherence (in the form of fragmentation and disorganization) in trauma memories of individuals with PTSD compared to healthy controls (e.g., Foa et al., 1995; Harvey and Bryant, 1999; Halligan et al., 2003; Jones et al., 2007; Kenardy et al., 2007; Jelinek et al., 2009, 2010; Salmond et al., 2011; or see Brewin, 2014 for a review). This incoherence also seems to predict the course of PTSD over and above initial symptoms (Engelhard et al., 2003; Halligan et al., 2003; Buck et al., 2007; Jones et al., 2007; Ehring et al., 2008). There are, however, numerous other studies that do not seem to support this hypothesis (e.g., Berntsen et al., 2003; Waters et al., 2013a,b; or see Rubin et al., 2016b for a review). Crucial differences between studies could possibly contribute to these inconsistent findings. Some studies focus for example on aspects of global coherence (i.e., life story coherence) whereas other studies look at local coherence of the trauma memory in particular. Variability in coherence ratings (self-report, observer, etc.) and small sample sizes could also add to these inconsistencies (Brewin, 2016).

### Summary and Discussion

Research on memory coherence has clearly demonstrated its importance for well-being and effective functioning of the self throughout the years. To recapitulate, memory coherence is associated with emotional problem solving, creating a personal identity, and establishing and maintaining a social network. Additionally, memory coherence has been found to be negatively associated with depressive symptoms, behavioral problems, and PTSD. Yet, there are some unresolved theoretical questions and inconsistencies, as well as methodological issues within this research field. First of all, there remains a lack of clarity about which subcomponent of memory coherence (i.e., context, chronology, or theme) is more or most important for the self. Since Reese et al. (2011) proposed their multidimensional model of coherence, there have been very few studies directly comparing these dimensions in terms of their association with well-being and psychopathology (Waters et al., 2013a; Rubin et al., 2016b). Furthermore, since the memory coherence literature has taken more of a developmental approach throughout the years, several remaining questions regarding the dynamics between coherence and psychopathology remain present. There is, for example, little information about the nature and the direction of the relationship between memory coherence and psychopathology, since most studies are cross-sectional in nature. It remains therefore unclear whether memory incoherence is truly a predictor of psychopathology or a mere by-product of the disorder. Additionally, knowledge about possible underlying mechanisms that could explain the relationship between memory coherence and psychopathology is rather scarce. More insight into the dynamics between memory coherence and psychopathology holds great potential for clinical practice (e.g., screening at-risk individuals, training memory coherence as treatment for certain disorders), but more research is needed. Besides unresolved theoretical questions, there are some methodological issues. Memory coherence has been operationalized in various ways throughout the years. Additionally, different research fields that study memory coherence, have used different coding schemes to assess coherence within personal narratives. This lack of clarity about the definition and assessment of memory coherence makes it challenging to directly compare studies across research fields. Unity and agreement in this regard could enhance our understanding of memory coherence by making it possible to directly compare studies and by enabling studies to build directly on each other.

## Toward an Integration

Memory specificity and memory coherence are both features of autobiographical memories that, as we portrayed earlier, relate to aspects of well-being and effective functioning of the self. Research on these two features developed throughout the past few decades as two largely independent research domains, with a more cognitive and psychopathological approach to the study of memory specificity and a more developmental approach to memory coherence. This separate development is unfortunate given the fact that memory specificity and memory coherence show a great number of similarities. These similarities made us wonder how these two features of autobiographical memory relate to each other. However, to the best of our knowledge, there have been no studies examining the relationship between these two features of autobiographical memory. Insights regarding this association are important from a theoretical and a clinical point of view. Evidence for an association between memory specificity and memory coherence would for example allow for a more extensive empirical and theoretical integration of these two domains in the future. Knowledge from 30 years of research on memory specificity could offer insights in the study of memory coherence (e.g., the work on the dynamics between autobiography and psychopathology, the work on training autobiographical memory specificity as a treatment protocol for depression). Conversely, the work on memory coherence, which has a strong developmental background, would provide a broader framework in which the work on memory specificity could potentially be fitted. Both domains have their own strengths and weaknesses, and both could potentially benefit from integration by taking advantage of the other’s strengths. In this section, we will link memory specificity and memory coherence by discussing some important similarities between the two and by formulating hypotheses about how they might relate to each other. We will make a first attempt at a theoretical integration of memory specificity and memory coherence by situating them in the Self-Memory System of Conway and Pleydell-Pearce. We will conclude this article by suggesting some new and exciting research possibilities and by explaining how both research fields could benefit from integration in future research.

### Similarities between Memory Specificity and Memory Coherence

There seems to be quite some overlap between the way memory specificity and memory coherence are operationalized, especially between specificity and contextual coherence. One of the criteria to categorize a memory as specific according to the AMT, is that the memory has to consist of one particular event that happened at a particular time and place (Williams and Broadbent, 1986). This notion of time and place forms the basis of contextual coherence (see Reese et al., 2011). We would therefore expect that memory specificity and contextual coherence would be positively related.

Memory specificity and memory coherence also show a quite similar developmental pathway throughout childhood and adolescence. Young children are generally less specific and coherent than older children, although they are capable of creating specific and coherent stories when parents guide them by asking specific questions (O’Carroll et al., 2006; Reese et al., 2011). Specificity and coherence both increase with age due to the development of a similar skillset that supports specificity and coherence, such as overall verbal abilities, notion of time and place, perspective taking, memory storage capacity, and so on (Howe and Courage, 1997; O’Carroll et al., 2006; Reese et al., 2011). Memory specificity, as well as chronological and contextual coherence, are largely developed by middle childhood (Gathercole, 1998; O’Carroll et al., 2006; Reese et al., 2011). Thematic coherence requires more sophisticated abilities like self-knowledge and insight, and develops further throughout adolescence (Reese et al., 2011).

As we mentioned earlier, autobiographical memory serves the self in different ways; through self-guidance, self in relation, self-identity, and self-regulation. Both memory specificity and memory coherence are involved in these associations in similar manners. Both support effective problem solving, be it in a slightly different manner. Recalling specific past experiences that resemble the current situation enhances problem solving by transferring knowledge about the way the previous situation was solved to the current situation (Evans et al., 1992; Goddard et al., 1996, 1997; Scott et al., 2000; Raes et al., 2005). Additionally, the ability to create a coherent narrative about the current situation makes it easier to work through the emotions evoked by the situation (Pennebaker, 1997; Fivush and Baker-Ward, 2005; Fivush et al., 2008). One could then utilize this previous experience for future occurrences. So, memory specificity facilitates practical problem solving, whereas memory coherence promotes the process of emotional problem solving.

In addition to being involved in problem solving, memory specificity and memory coherence both play a role in the development of a personal identity. As we pointed out earlier, creating a sense of self is a crucial developmental task during adolescence (Erikson, 1968; McAdams, 1985). By autobiographical reasoning, the adolescent will link together different specific memories that are unique to the individual and differentiates him- or herself from others. By interpreting, evaluating, and connecting these single memories, the adolescent is able to form a coherent life story, which is a subjective representation of one’s personal development (Bluck and Habermas, 2000; Habermas and Bluck, 2000; Habermas and de Silveira, 2008; Fivush et al., 2011). Creating a coherent life story is key to a healthy identity development and leads to feelings of continuity, purpose, and meaning (Kernberg, 1984; Antonovsky, 1985; Bluck and Habermas, 2000; Habermas and Bluck, 2000; Habermas and de Silveira, 2008).

Autobiographical memory also serves a social function. Through reminiscing with others about personal experiences, our lives become intertwined and we create a shared past (Fivush et al., 2006). The way in which this reminiscing occurs, especially in the context of mother-child reminiscing, relates to the development of both memory specificity and memory coherence. Mothers who are more elaborate when reminiscing with their children about their personal experiences (e.g., playdate or school trip) tend to have children who are more specific and coherent, concurrently and over time (Fivush and Fromhoff, 1988; Valentino, 2011; McDonnell et al., 2016). Elaborating on the circumstances surrounding the experience (e.g., cause, context, consequences) and the feelings it evoked (i.e., structural and emotional elaboration) facilitates children’s memory specificity by helping them form a coherent narrative of their emotional experiences (Fivush, 2011; McDonnell et al., 2016). Whether elaboration positively predicts memory specificity and memory coherence depends on mother–child attachment status. Mothers are usually more elaborate when the child is securely attached (Fivush and Vasudeva, 2002). In contrast, elaborative reminiscing predicts less memory specificity in anxious or avoidant attached children (McDonnell et al., 2016). So, in addition to showing similar developmental pathways, the development of memory specificity and memory coherence are both influenced by the same contextual characteristics that can account for individual differences. Both features of autobiographical memory are scaffolded by elaborative mother-child reminiscing, and this association seems to be moderated by child attachment status.

In the previous two sections, we discussed how both memory specificity and memory coherence relate to the self in terms of self-regulation and psychopathology. This overview showed that difficulty recalling specific memories and difficulty creating coherent narratives about personal experiences show highly similar associations to psychological disorders. Both overgeneral autobiographical memory and memory incoherence are related to the presence of depressive symptoms and PTSD. As we pointed out earlier, besides inconsistencies in the literature, several questions concerning the relationship between memory coherence and internalizing symptoms and disorders remain unanswered. Later on, we will explain in more detail how an integration could potentially contribute to resolving some of these inconsistencies and unresolved questions.

Besides these similarities, there are some conditions in which memory specificity and memory coherence seem to differ from one another. For example, episodic amnesia seems to impact memory specificity and memory coherence differently. Whereas patients suffering from episodic amnesia show deficits in recalling specific past events, they are still able to create coherent narratives if sufficient details are provided to them. So, it seems that episodic amnesia interferes with generating details about events instead of binding them into a coherent narrative, suggesting that damage to the hippocampus may influence the ability to recall specific events differently than the ability to create coherent narratives (Keven et al., 2017). To fully grasp the relationship between memory specificity and memory coherence, more research seems necessary to further explore the conditions in which they relate to each other and the conditions in which they seem to differ.

### Integration within the Self-Memory System

Within the Self-Memory System, personal memories are stored across four different hierarchical layers going from abstract and general to concrete and specific (i.e., life story schema, lifetime periods, general events, and event-specific knowledge). As a first attempt to integrate memory specificity and memory coherence, we will situate them both within this theoretical model. This will provide more insight about how they might relate to each other and to the self, which can guide future research.

When trying to position complex cognitive phenomena like memory specificity and memory coherence in a theoretical model, we have to make certain assumptions. Both memory specificity and memory coherence are measured by scoring written or oral responses to cue words or instructions. These responses don’t necessarily tell us something about how this autobiographical knowledge is organized on a higher cognitive level, since we cannot access this directly. Therefore we assume that someone’s ability to retrieve specific events and create coherent accounts of the events is a reflection of the way this personal information is organized within the individual’s memory. However, whether or not this assumption is a correct representation of the reality opens up a whole other debate, which falls beyond the scope of this manuscript.

Memory specificity represents a subclass of memories about single experiences that did not last longer than 1 day and therefore corresponds with the level of event-specific knowledge, which is the most specific and concrete level in the Self-Memory System. Overgeneral autobiographical memory arises when the search for specific memories truncates at a higher, more general and abstract level (Conway and Pleydell-Pearce, 2000; Williams et al., 2007). Different processes can influence this search and contribute to the development of overgeneral memory (Williams et al., 2007).

As we mentioned earlier, for a memory to be situated at the most specific level in the Self-Memory System, it must refer to a single event and it has to be rich in sensory and perceptual detail. These requirements for detail fit well with the criteria for memory coherence. A coherent memory contains specific information about where and when the event took place (i.e., context) and how the event unfolded (i.e., chronology). Additionally, the memory contains subjective information such as the emotions and cognitions that were evoked by the experience (i.e., theme). Although the focus typically rests on a single event, coherent memories are often extended in time. The individual will, for example, situate the event in his life story and describe the precursors and the consequences of the event, both short and long term. If this were to be the case when coding for memory specificity, such a memory would be categorized as a general memory because the experience described lasts longer than 1 day. However, Conway and Pleydell-Pearce do not impose a time restriction, therefore allowing memory coherence to be situated at the level of event-specific knowledge. So, we hypothesize that both memory specificity and memory coherence can be situated at the same hierarchical level in the Self-Memory System.

Positioning memory coherence at the most specific level of the Self-Memory System raises additional hypotheses about the relations between memory coherence, memory specificity, and the self. As we mentioned earlier, memory coherence is involved in emotional problem solving through the process of meaning making. Negative or stressful experiences are usually in conflict with the individual’s current goals. The individual will strive to resolve this discrepancy between the current situation and current goals. By creating a coherent narrative about the negative experience, the individual will be able to create meaning and come to some sort of resolution (e.g., the realization that the current experience is not as negative as originally thought, changing goals to better fit the current circumstances, etc.), making the negative experience reconcilable with the working self. Additionally, placing memory coherence within the Self-Memory System makes it possible to theorize about how the coherence of single experiences relates to the overall coherence of the life story. The latter is represented by the life story schema, which is situated at the top layer of the Self-Memory System. As we mentioned earlier, being able to create a coherent overall life story is key to a healthy identity development. Through autobiographical reasoning, specific and coherent memories will be connected and integrated into a coherent life story schema. The working self (which contains a hierarchy of current goals) and the conceptual self (which contains personal attitudes, beliefs, etc.) will determine which of those event-specific memories will be integrated and stored within the life story schema. This shows how memory specificity, memory coherence, and global life story coherence interact to form a stable sense of self.

For this integration section, we based ourselves on Conway and Pleydell-Pearce’s Self-Memory System, which offers a potential framework to look at the relationship between memory specificity and memory coherence and how they relate to the self. However, the Self-Memory System is just one way of trying to integrate memory specificity and memory coherence and does not provide absolute or definite answers. Nonetheless, this attempt at an integration suggests that there are a lot of open questions regarding the relationship between memory specificity, memory coherence, and the self that can and should be investigated in future research.

### Future Research Recommendations

As we pointed out throughout the previous sections, there is still room for improving our understanding of memory specificity and memory coherence. We argue that one possible way to achieve this is by examining how these features of autobiographical memory relate to each other and how they relate to the self. Given the similarities between memory specificity and memory coherence, we hypothesize that at least a moderate positive association could be expected between them. It seems indeed reasonable to assume that if a person has only limited access to specific personal memories, this would strongly impede the construction of coherent narratives about these experiences. Difficulty retrieving specific personal memories could also hinder the construction of a coherent life story, which requires the integration of multiple specific memories. Conversely, if an individual cannot make sense of an experience, and therefore cannot create a coherent narrative, this could disturb the emotional processing of the event, which could make retrieval of specific memories too threatening (Todd et al., 2013). This could in turn make the individual vulnerable for the development of emotional disorders. Additionally, given the similarities between memory specificity and contextual coherence, it is possible that memory specificity in fact is a subcomponent of memory coherence. Although we cannot make any definite claims about how memory specificity and memory coherence relate to each other, these different hypotheses demonstrate that further research is necessary.

More specifically, integrating memory specificity and memory coherence in future research could potentially provide answers to some remaining questions or resolve inconsistencies. For example, it is unclear whether memory incoherence is a vulnerability factor for the development of internalizing symptoms or a mere consequence or by-product of those symptoms. Likewise, little is known about possible underlying mechanisms that can explain this relationship. Research on memory specificity has focused greatly on its association with psychopathology, resulting in different theoretical models attempting to explain this association. Integrating memory specificity and memory coherence could therefore offer insights into the dynamics between memory coherence and psychopathology. For instance, the mechanisms described in the CaR-Fa-X model could be evaluated for memory coherence to get a better understanding of how and why incoherence relates to the presence of psychopathology. Additionally, research has shown that overgeneral autobiographical memory is not a general characteristic of psychopathology. It is, for example, related to the presence of depression, but not to anxiety-related disorders. It is possible that the same applies to memory coherence, which could potentially resolve the inconsistencies we pointed out earlier. Internalizing symptoms form a rather heterogeneous group of symptoms (i.e., depression and anxiety-related symptoms) and it is possible that memory incoherence only relates to the presence of one of these two, which could explain the inconsistent findings. Future research should therefore examine how memory coherence relates to the presence of depressive versus anxiety-related symptoms by using specific instruments instead of broadband screening questionnaires that do not differentiate between different types of internalizing symptoms. So, by transferring knowledge about the dynamics between memory specificity and psychopathology, we could potentially resolve certain inconsistencies regarding the relationship between memory coherence and psychopathology.

The domain of memory specificity could likewise benefit from an integration in future research. As we mentioned earlier, there seems to be some uncertainty regarding what is actually being measured with the AMT. A strong correlation between memory specificity and contextual coherence could indicate that memory specificity can be seen as a component of memory coherence, placing specificity within a broader framework. Insight into the relation between memory specificity and memory coherence could also have some clinical implications. For example, Memory Specificity Training (MEST) has been shown to be effective in decreasing depressive symptoms (Raes et al., 2009). If memory specificity would indeed be a part of the coherence construct, this could influence the content of the memory training. Instead of only focusing on specificity, the training could target overall memory coherence.

## Conclusion

Research on memory specificity and memory coherence has developed as two largely independent research domains over the past three decades. This is, in our opinion, quite surprising since they both show highly similar associations to well-being and effective functioning of the self. These similarities make us therefore wonder how these two features of autobiographical memory relate to each other. Integration of these research domains offers new and exciting possibilities for future research and could result in new insights that enhance our understanding of the relationship between autobiographical memory and the self.

## Author Contributions

The manuscript was written by EV after frequently brainstorming with DH, who also critically revised the article on multiple occasions. Likewise, PB contributed to this manuscript by revising the article and giving feedback.

## Conflict of Interest Statement

The authors declare that the research was conducted in the absence of any commercial or financial relationships that could be construed as a potential conflict of interest.
